# Queer Industries in New York

**Published:** 1881-04

**Authors:** 


### QUEER INDUSTRIES IN NEW YORK.

The investigations of the census men have led to some queer developments in the manufactories of New York and adjoining cities. The largest single industry in New York is that of custom-made clothes. The making of paper patterns employs hundreds of hands, and, ten houses being engaged in it, uses tons of paper. There are factories for making dried blood, the dummies that milliners use to show dresses on, theatrical armor and jews-harps. The use of adulterating substances is getting to be general. "Castile soap" is made of grease and terra alba, or white earth, which earth is also used largely in candy making. Glucose, which is corn syrup, is used heavily by the sugar refiners. There is a firm engaged in making honey and honey-comb. The honey-comb is made by machinery, of paraffine wax, and is an exact imitation of the regular thing, except that the bees fashion their cells of walls only 1-125th of an inch wide, while human artificers have not yet become that deft. The cells are filled with glucose, which is the sweet syrup of common corn, and looks and tastes like honey. The cells, once filled, are closed by smearing a hot iron plate over the wax tops, and the product is sold as the "best clover honey." It is in great demand, and out-sells the regular honey. Gallons and gallons of the best tomato catsup are made from the tomato skins which are purchased from the great tomato canning establishments.—*Correspondence Atlanta Constitution.*

A GERMAN was in a room with a dozen other lodgers, trying to sleep, but was kept awake by their terrific snoring. At last one of the snorers, who had been shaking the building for half an hour, gave a snort and stopped short. "Tank Gott, von ish tead!" said the Dutchman.

